# Going Mobile: Using Portable Genomic Technologies for PCR‐Free In Situ Species Identification and Real‐Time Molecular Systematics

**DOI:** 10.1002/ece3.72442

**Published:** 2025-11-05

**Authors:** Evan J. Kipp, Marissa S. Milstein, Lexi E. Frank, Roxanne J. Larsen, Tiffany M. Wolf, Christopher Faulk, Christopher A. Shaffer, Peter A. Larsen

**Affiliations:** ^1^ Department of Veterinary and Biomedical Sciences, College of Veterinary Medicine University of Minnesota St. Paul Minnesota USA; ^2^ Department of Fisheries, Wildlife and Conservation Biology, College of Food, Agricultural and Natural Resource Sciences University of Minnesota St. Paul Minnesota USA; ^3^ Priogen Corp St. Paul Minnesota USA; ^4^ Department of Veterinary Population Medicine, College of Veterinary Medicine University of Minnesota St. Paul Minnesota USA; ^5^ Department of Animal Science, College of Food, Agricultural and Natural Resource Sciences University of Minnesota St. Paul Minnesota USA; ^6^ Department of Anthropology Grand Valley State University Allendale Michigan USA

**Keywords:** adaptive sampling, Chiroptera, Culicidae, mitogenome, Phlebotominae, phylogenetic capture

## Abstract

Across the globe, anthropogenic environmental changes are threatening animal biodiversity and contributing to the emergence of vector‐borne and zoonotic pathogens through host range shifts. To combat these challenges, accurate and timely biodiversity assessments and molecular species monitoring efforts are critical. Here, we document how the implementation of a portable laboratory in combination with targeted long‐read nanopore sequencing can facilitate in situ genomic and systematic analyses across several animal taxa. Working at two ecologically divergent field sites in Guyana, South America, we collected small mammals and blood‐feeding insects, including bats, rodents, a marsupial, mosquitoes, and a phlebotomine sand fly. For each specimen sampled, genomic DNA was extracted in the field and used for the preparation of nanopore sequencing libraries. For field sequencing, we utilized a novel software‐based targeted sequencing approach—nanopore adaptive sampling (NAS)—that enabled the selective sequencing of mitochondrial reads using mitogenome assemblies of related taxa as enrichment targets. Basecalled reads from our field sequencing experiments were used to assemble complete mitogenomes and to generate mitochondrial biomarker consensus gene sequences for all nine small mammals and four blood‐feeding insects sequenced. Confirmatory molecular identifications were made with a combination of local nucleotide BLAST queries and maximum likelihood analyses using biomarker consensus sequences. Importantly, the mitogenome‐based targeted sequencing strategies outlined here are amplification‐free and allowed us to bypass time‐consuming and potentially troublesome PCR‐based methods in the field, streamlining library preparation, sequencing experiments, and on‐site analyses. Our findings describe targeted sequencing with NAS as an effective tool for implementation into portable laboratories to widely enhance field‐based biodiversity monitoring and rapid molecular species assessments across vertebrate and invertebrate hosts of consequential emerging pathogens.

## Graphical abstract

Here, we document how the implementation of a portable laboratory, in combination with targeted long‐read sequencing through an approach known as nanopore adaptive sampling, can facilitate in situ genomic and systematic analyses across a wide range of animal taxa, including multiple small mammals and blood‐feeding arthropods. Through experiments performed at two ecologically diverse field sites in Guyana, South America, we performed targeted mitochondrial genome sequencing to generate on‐site molecular species identifications. Our findings describe targeted sequencing with nanopore adaptive sampling as an effective tool for implementation into portable laboratories to widely enhance field‐based biodiversity monitoring and rapid molecular species assessments across vertebrate and invertebrate hosts of consequential emerging pathogens.

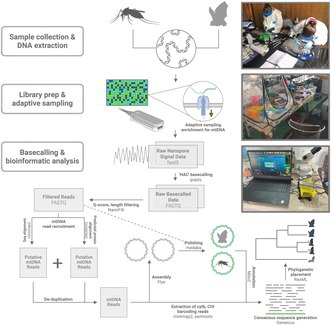

## 1 Introduction

Earth's ecosystems are being rapidly reshaped by climate and land‐use changes, driving shifts in animal ranges with far‐reaching consequences for biodiversity conservation and the transmission of emerging zoonotic and vector‐borne diseases (Bellard et al. 2012; Carlson et al. 2022; Festa et al. 2023; Osland et al. 2021). In light of these ongoing global changes, accurate and timely biodiversity assessments are essential for documenting species presence or absence at particular points in time. Beyond census data, measures of biodiversity are used to characterize baseline species assemblages, establish wildlife‐protected areas, and analyze population and functional genomics for wildlife management, all of which ultimately inform conservation priorities and policies (Formenti et al. 2022; Marsh and Trenham 2008; Robinson et al. 2018; Scheele et al. 2019). Moreover, such biodiversity studies are significantly strengthened with the incorporation of molecular systematic data. When gathered alongside field observations and morphologic data, these molecular data can help elucidate evolutionary trajectories, biogeographic patterns, and hybridization events (Bik et al. 2012; Creer et al. 2016; P. A. Larsen et al. 2010; Malukiewicz et al. 2021; Pennisi 2019). For this reason, methodologies that incorporate biodiversity monitoring with molecular systematic analyses (e.g., phylogenetics, phylogeography) that are globally accessible, and do not require a priori knowledge of sampled species are of particular interest. Traditionally, field‐based species identification has relied on morphological assessment alone. This approach remains challenging, as it is time‐consuming and often requires a taxonomic specialist to differentiate between subtle morphological features of cryptic species (Beebe 2018; Novaes et al. 2024; Pante et al. 2015). For some specimens, morphological species identification can take months or years to achieve.

Advancements in molecular systematics, particularly through the use of DNA barcoding and metabarcoding, have been critical for accelerating species identification and testing evolutionary hypotheses (Creedy et al. 2021; Creer et al. 2016; Hebert and Gregory 2005; Janzen et al. 2009; Jinbo et al. 2011). DNA barcodes, or DNA biomarkers, are typically short ~400–700 bp fragments of DNA that contain sequences of diagnostic utility for species rank assessments when compared against a reference database. The mitochondrial genome (mitogenome) is commonly used for gene‐specific molecular barcodes given its higher per‐base mutation rate in comparison to the nuclear genome (Boore 1999). As a result, mitochondrial genes evolve rapidly during speciation events, permitting identification of species‐level differences based on nucleic acid or amino acid sequence identity (Cameron 2014; Gissi et al. 2008). In animals, the most commonly used DNA biomarkers consist of mitochondrial gene sequences of the cytochrome c oxidase I (*COI*) gene and cytochrome‐b (cyt*b*) gene (Bradley and Baker 2001; Hebert, Cywinska, et al. 2003; Hebert, Ratnasingham, et al. 2003). When combined with morphologically defined taxonomic units (e.g., genera, species, subspecies) and geographic sampling efforts, phylogenetic analyses of mitochondrial DNA barcodes have routinely informed molecular systematic studies of numerous animal taxonomic groups (Janzen et al. 2009; Jinbo et al. 2011; Pennisi 2019). The use of DNA barcodes has also led to the development of large‐scale databases such as the Barcode of Life Data System (BOLD) that can be leveraged for species‐level biodiversity classification (Ratnasingham and Hebert 2007).

Biodiversity hotspots in the tropics are often located in countries that do not have the laboratory infrastructure required for molecular species identification (Antonelli et al. 2018; Jones et al. 2008; Pomerantz et al. 2018). High‐throughput sequencing traditionally requires large, permanent, laboratory equipment and specialized expertise that can be cost‐prohibitive. Moreover, it is becoming increasingly difficult to conduct biodiversity research that relies on the exportation of biological samples, as country‐specific permitting processes can be challenging to navigate and there are ethical considerations of removing samples from their country of origin (Ambler et al. 2021; Colella et al. 2023; Martinez et al. 2020; Ramírez‐Castañeda et al. 2022).

For the reasons discussed above, there is a need for accessible genomic technologies that are globally deployable and facilitate in situ biodiversity research. Fortunately for field researchers, a variety of portable molecular instruments are now readily available and are becoming increasingly reliable, rugged, and affordable (Chen et al. 2023; Edwards et al. 2022; Watsa et al. 2020). Miniature PCR and real‐time PCR thermal cyclers (e.g., MiniPCR, Biomeme, BMS Mic) and single‐molecule nanopore sequencers (Oxford Nanopore Technologies Inc. (ONT) MinION Mk1b and Mk1c instruments) can now be combined alongside other field‐optimized components into a portable laboratory, or mobile lab, capable of generating on‐site molecular data anywhere in the world. The use of such mobile labs has enabled a range of molecular analyses over diverse field settings including Madagascar (Blanco et al. 2020), Antarctica (Johnson et al. 2017), Ecuador (Chaves et al. 2023; Pomerantz et al. 2018), and the International Space Station (Castro‐Wallace et al. 2017; Stahl‐Rommel et al. 2021).

Here, we show how portable genomic technologies and downstream systematic analyses can be used for real‐time in situ molecular identification of potential pathogen hosts and vectors at two remote field sites in Guyana, South America. While previous portable laboratory studies have focused exclusively on PCR‐based approaches, we document the field use of a novel strategy for targeted and amplification‐free sequencing: nanopore adaptive sampling (NAS). Using ONT's MinION sequencing instruments and a computer equipped with a robust graphics processing unit (GPU), NAS maps nucleotides against a reference database while individual reads are being basecalled in real‐time (Payne et al. 2021). Based on user‐defined thresholds, reads can be rejected or retained by sequencing nanopores while they are being sequenced, providing considerable downstream enrichment in the resulting sequencing output toward specified genomic targets (De Meulenaere et al. 2024; Martin et al. 2022; Sun et al. 2023). In our evaluation of NAS for field‐based molecular species barcoding, we outfitted a mobile lab capable of DNA/RNA extraction, nanopore library preparation, and DNA sequencing with NAS. Informed by recent work demonstrating that NAS‐based ‘phylogenetic capture’ methods are effective at facilitating rapid, PCR‐free identification of mammal and insect species (Frank et al. 2024; Kipp et al. 2023; Wanner et al. 2021), we elected to conduct sequencing through NAS using whole mitochondrial genomes as enrichment references. We hypothesized that NAS would allow for targeted capture of mitochondrial DNA (mtDNA) reads in specimens sequenced in the field to enable mitogenome and biomarker gene assemblies for use in phylogenetic analyses for molecular species identification. Our findings suggest that NAS can serve as a particularly useful tool for integration into portable laboratories and field sequencing workflows aimed at rapid biomonitoring and in situ species barcoding.

## 2 Materials and Methods

### 2.1 Small Mammal and Insect Collection

**FIGURE 1:**
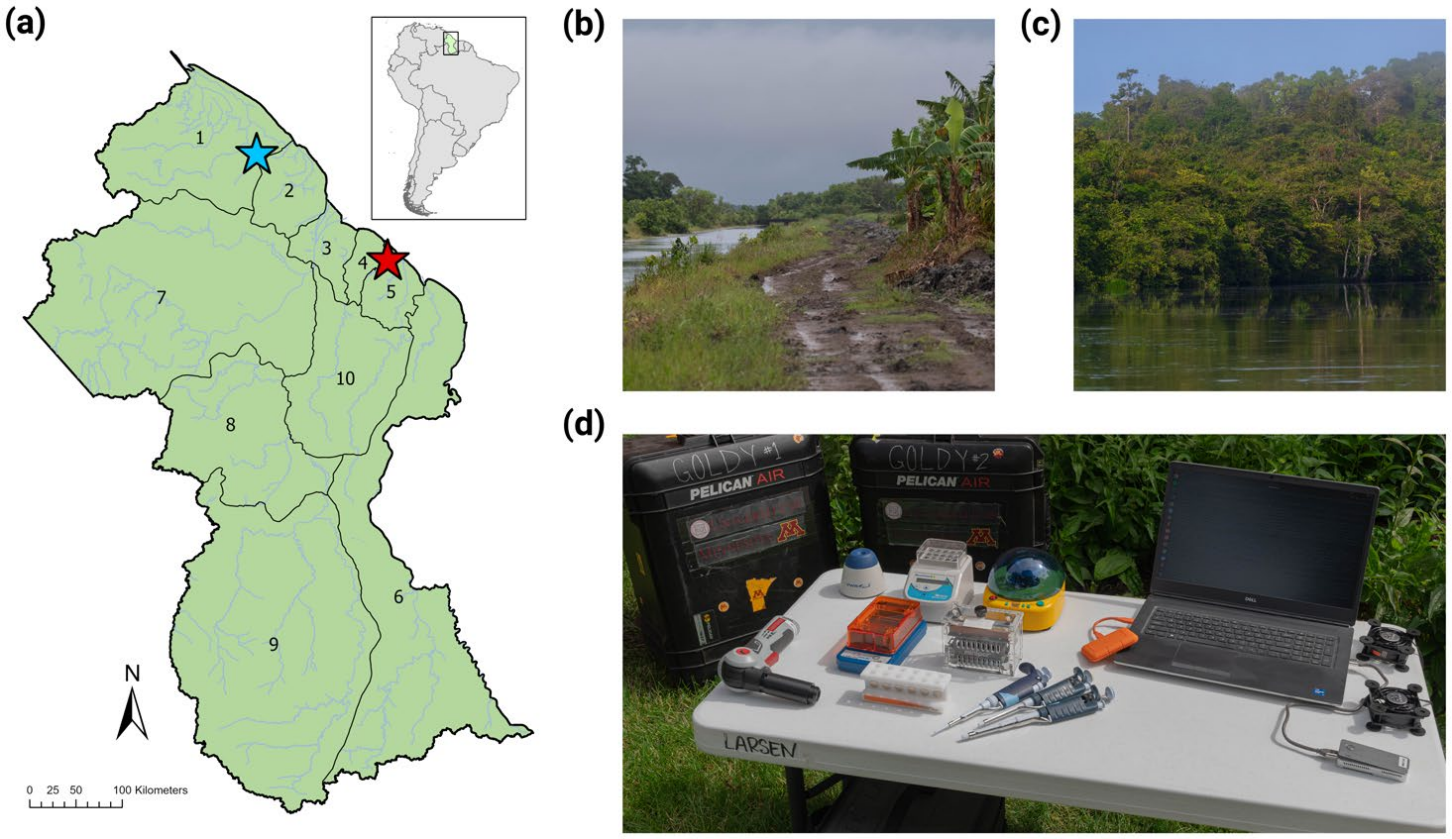
Sites for field sequencing experiments and portable laboratory equipment used. (a) Map of Guyana, South America with regional boundaries and locations of the Mahaica (red star) and Kwebana (blue star) study sites. (b) Representative photo of the heavily disturbed and agricultural landscape surrounding the Mahaica site. (c) Secondary forest at the Kwebana site. (d) Key pieces of portable lab equipment used for field DNA extractions, nanopore library preparation, and sequencing experiments.

Small mammal and insect collections were conducted across two study sites in Guyana, South America between June 5 and June 21, 2022 (Figure 1a). One site, Mahaica, is an agricultural community located in Region 4 (Demerera‐Mahaica) along the Mahaica river, approximately 25 miles from the capital city, Georgetown. This area has long been an important rice‐producing region of Guyana. This field site is characterized by high levels of human disturbance and intensified agriculture, with extensive permanent rice fields and small, highly isolated forest fragments (Figure 1b). The second site, Kwebana, is an Indigenous village of approximately 2000 people located in the Northwest of Guyana in Region 1 (Barima‐Waini). The village is a sprawling community of about six miles along the Kwebana‐Santa Rosa road. The area surrounding the community contains a mosaic of habitat types, including primary and secondary forest fragments and both permanent and swidden agricultural fields (Figure 1c). In the past 30 years, the area around Kwebana has experienced considerable forest degradation because of increased population, commercial logging, agriculture, and gold mining. Across both sites, and particularly within the Indigenous community at Kwebana, collection of samples and following mobile lab molecular experiments were performed with the full approval and support of community leadership, and with a focus from our group on promoting local teaching and providing community members with explanations of all research findings.

Small mammals—which included bats, rodents, and marsupials—were captured, humanely euthanized, and necropsied at both study sites. Over a total of 10 trap nights, we sampled bats using mist nets, placing nets near homes and other peridomestic areas, potential flyways, rivers, fruiting trees, and adjacent to possible roost sites to target a wide diversity of bat taxa (Trevelin et al. 2017). We focused rodent and marsupial trapping on peridomestic species using Sherman traps placed in and around houses and buildings over six total nights of trapping. All small mammal trapping, handling, and euthanasia methods were conducted following protocols approved by the University of Minnesota IACUC, under protocol number 2105‐39114A, and adhering to the American Society of Mammalogists guidelines for humane euthanasia (Sikes and The Animal Care and Use Committee of the American Society of Mammalogists 2016). For all small mammals collected, we recorded body measurements, sex, age, reproductive status and additional metadata including location and date of capture. Field‐based morphologic species identification was performed following regional keys (Gardner 2019; López‐Baucells et al. 2018; Patton et al. 2020). Small mammal tissues collected during necropsies were immediately submerged in DNA/RNA Shield (Zymo Research, Irvine, United States). All tissues not selected for field DNA extractions were snap‐frozen in a liquid nitrogen dewar and accessioned for long‐term storage within the collections of the Natural Science Research Laboratory at the Museum of Texas Tech University. Whole‐body voucher specimens for collected mammals were fixed in a 10% buffered formalin solution and deposited within the Texas Tech University collections.

Insect sampling targeting potential pathogen vectors was additionally carried out at each study site using CDC miniature light traps and handheld aspirators (John W. Hock Company, Gainesville, United States). We installed light traps nightly over a total of five trap nights at the Mahaica site and seven trap nights at the Kwebana site. Light trap collection chambers were gathered the following morning and collected blood‐feeding insects, including mosquitoes and phlebotomine sand flies, were sorted from non‐target bycatch. Using available keys (Lane 1953), mosquitoes were identified to the genus or species rank, as possible, using morphologic characters through examination under a stereomicroscope. Whole insect specimens were preserved in DNA/RNA Shield and stored at ambient temperature prior to DNA extraction.

### 2.2 Portable Lab Equipment and DNA Extractions

Genomic DNA (gDNA) extractions and following molecular methods were performed in the field using a portable lab outfitted with instruments optimized for use in field or semi‐field (i.e., field sites with reliable or intermittent access to electricity) settings (Figure 1d). Lists and cost estimates of portable instruments and consumable materials are presented in Tables S1, S2, respectively. At each site, we derived electricity to operate the portable lab from gasoline‐powered generators, using solar‐powered batteries and supplemental battery packs as needed to power small devices (e.g., microcentrifuge, heat block). To minimize the potential for sample contamination, a small ‘pop‐up’ tent with clear vinyl siding was assembled to perform extractions and preparation of nanopore sequencing libraries. Throughout sample processing, we routinely sterilized interior and exterior tent surfaces, including pipettors and other instruments, with 70% ethanol.

For nucleic acid extractions, gDNA was isolated from roughly 25 mg of small mammal liver tissue or whole insect specimens using the DNeasy Blood and Tissue Kit (QIAGEN, Hilden, Germany) following manufacturer instructions. We elected to focus insect DNA extractions on a selection of cryptic specimens identified only to the genus level, which included predominant mosquito morphotypes observed at each study site. Preference for mammalian DNA extractions was given to cryptic specimens and those collected specimens that were not readily identifiable to the species rank using morphology alone. To promote tissue lysis, we placed insect and small mammal tissue samples in a solution containing 180 μL ATL lysis buffer and 20 μL Proteinase K. To promote digestion of insect exoskeletons for tissue lysis, insects were further crushed using disposable DNase/RNase‐free pestles. Tissue homogenates were then transferred to clean 1.5 mL collection tubes and were incubated on a heat block at 56°C for 30 min before being transferred to spin columns for the remaining centrifugation and wash steps. Centrifugation of spin column and collection tubes was done using a Gyro Plus microcentrifuge (MiniPCR Bio, Cambridge, United States), near the device's maximum speed of 10,000–12,000 rpm. As needed, we increased centrifugation times up to roughly 5 min until we could confirm visually that all wash solution or eluate had passed through the DNeasy silica membrane. We eluted extracted gDNAs into a volume of 100 μL in nuclease‐free water and quantified with a Qubit 4.0 fluorometer using the 1X dsDNA high sensitivity assay kit (Invitrogen, Waltham, United States).

### 2.3 Library Preparation and Sequencing Specifications

Nanopore libraries were prepared using ONT Sequencing Ligation Kit SQK‐LSK109 following the manufacturer's instructions for sequencing on either a R9.4.1 MinION (FLO‐MIN106D) or R9.4.1 Flongle (FLO‐FLG001) flow cell. Depending on the flow cell type selected and the initial count of available pores, we sequenced samples individually or in multiplex using ONT Native Barcoding Expansion Kit EXP‐NBD114. Library preparation followed steps outlined in the SQK‐LSK109 protocol with minor modifications and using additional reagents from the NEBNext Companion Module and the Blunt/TA Ligase Master Mix (New England Biolabs, Ipswitch, United States) for ligation reactions. We used a large hard‐sided cooler chilled with cold packs for transport of all ligases and library preparation kit reagents to each field site. Initial end‐prep and DNA repair reactions were performed using a mini16 thermal cycler (MiniPCR Bio, Cambridge, United States) to reach the 65°C incubation temperature; subsequent barcode and adapter ligation reactions were incubated at ambient temperatures.

We loaded all prepared libraries onto ONT MinION flow cells (FLO‐MIN106D), with the exception of a single bat sample (TK217562) which was loaded and sequenced on a reduced throughput, single‐use Flongle flow cell (FLO‐FLG001). Up to two libraries were sequenced per MinION flow cell, with a nuclease wash step performed between uses using ONT kit EXP‐WSH004. Sequencing was initiated through the MinKNOW software (ONT; v22.03.6) and performed on a Linux laptop computer running Ubuntu 18.04 and with the following hardware components: 16× 11th Gen Intel Core i7; Nvidia GeForce RTX 3080 Ti laptop graphics card with 16 GB video memory; 1 TB internal solid‐state drive. As both field sites lacked reliable internet, MinKNOW configuration settings were manually edited to allow for offline functionality. Specifications for each sequencing run were selected within MinKNOW and the adaptive sampling option was enabled. The NAS approach allows for real‐time enrichment of user‐defined sequences during an experiment (Payne et al. 2021), provided that the computer used for sequencing is equipped with a GPU capable of keeping up with live basecalling under the ‘fast’ basecalling configuration model. To target mtDNA read enrichment across our sampled taxa, we elected to use enrichment targets that included the complete mitochondrial genomes for both mammals and insects, obtained through the National Center for Biotechnology Information (NCBI) Organelle Genome Resources webpage (NCBI RefSeq accessed on May 17, 2022). Reference mitogenomes were downloaded locally, concatenated into a single FASTA file and converted to .mmi format using the minimap2 index function (H. Li 2018). After initiation, we allowed sequencing runs to continue overnight or until it appeared that data output from the run had plateaued, and libraries were near fully sequenced.

After completion of each sequencing experiment, raw FAST5 files were re‐basecalled with ONT's guppy basecaller (v6.0.7). Here, we elected to use the ‘high accuracy’ or ‘HAC’ basecalling configuration model (dna_r9.4.1_450bps_hac.cgf) for post hoc basecalling to improve basecall accuracy over ‘fast’ basecalled data generated live during the run. Although guppy also contains a ‘super accuracy’ model, the considerably longer processing time taken to basecall under the SUP model precluded it as an efficacious option in the field. Post hoc HAC‐basecalled data were output in FASTQ format and further demultiplexed and concatenated by sample. All basecalled field‐generated data are deposited on Dryad (https://doi.org/10.5061/dryad.bnzs7h4p2) alongside insect and small mammal mitogenome reference FASTA files used during NAS enrichment.

### 2.4 Preliminary Field‐Based Species Identification

**FIGURE 2:**
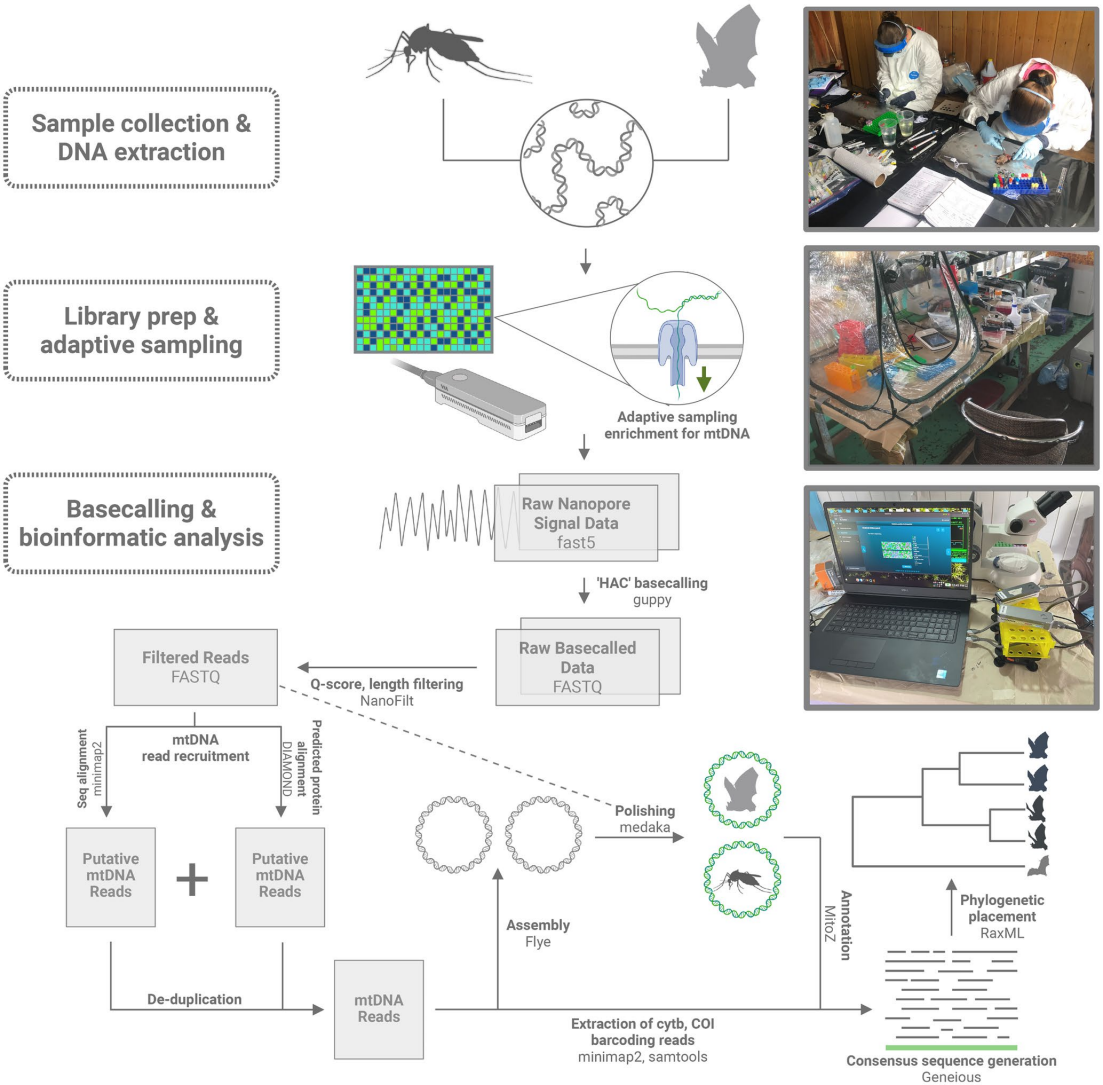
Summary of field sequencing methods and computational steps to process and analyze field‐generated sequence data. Tissue samples were collected (top photo) at each field site. After nucleic acid extraction, nanopore libraries were prepared with components of the portable lab (middle photo). Sequence data were generated and basecalled on‐site using adaptive sampling targeting mtDNA (bottom photo). The resulting data were quality filtered and mtDNA reads were extracted to generate de novo mitogenome assemblies, generate biomarker consensus sequences, and perform molecular systematic analyses. Inset photos taken by E.J.K.

Following post hoc basecalling, we implemented a rapid and field‐friendly analysis pipeline to provide putative species identifications utilizing an assortment of shell commands with common bioinformatic tools (Figure 2). A detailed list of commands and software packages used is provided in the Supporting Information. First, we quality and read‐length filtered basecalled data for each sample using the NanoFilt package (De Coster et al. 2018) with a q‐score cutoff value of 7. headcrop of 50 bp. Minimum and maximum read length filters were set between 300 bp and 17 kb to remove excessively short reads and any off‐target non‐mtDNA reads that escaped NAS rejection. Next, we mapped those filtered reads for each sample against the complete mitogenome RefSeq FASTA used as a reference during NAS enrichment using minimap2 (H. Li 2018). In parallel, protein‐based alignment using DIAMOND (v2.0.15) was also used to capture putative mtDNA reads via amino acid homology following previously described methods (De Vivo et al. 2022). Putative mtDNA reads from both DNA‐ and protein‐based alignment approaches were concatenated into a single FASTA and duplicate reads were removed using the dedupe.sh script from the bbmap package (Bushnell 2014). A local install of the basic local alignment search tool (BLAST; v2.5.0+) was used to perform a nucleotide BLAST (blastn) to query our putative mtDNA reads against an assortment of custom BLAST databases (e.g., the complete mitogenome RefSeq, individual COI and cytb databases for bats, rodents, and arthropods obtained through NCBI and BOLD databases). We performed these blastn queries in real time while sequencing was in progress and after completion of sequencing following post hoc HAC basecalling. After queries were completed, we examined top‐ranking read BLAST hits based on percent identity, query coverage, and e‐value to provide a preliminary species‐level identification prior to de novo assembly of mtDNA reads.

### 2.5 Mitogenome Assembly and Phylogenetic Analysis

Using isolated and de‐duplicated mtDNA read data sets for each sample, we performed de novo assembly using the flye software package with flags included for assembly from ‘high‐quality’ error‐corrected ONT reads and an estimated genome size of approximately 17 kb. Assemblies were manually inspected to identify and pull circular contigs roughly 15–17 kb in size consistent with complete animal mitochondrial genomes. Draft mitogenomes were subjected to a single round of polishing with medaka using the ‘medaka_consensus’ command and the ‘r941_min_hac_g507’ chemistry and basecalling model. Polished assemblies were annotated using MitoZ v.3.4 (Meng et al. 2019). The resulting mitogenomes with gene annotations were visualized in Geneious Prime software (v2022) and consensus sequences for phylogenetically informative protein‐coding genes were extracted for creating multiple sequence alignments. Alternatively, for samples for which only a portion of the mitogenome was assembled, we utilized an alternate strategy in which de‐duplicated mtDNA reads were not assembled de novo, but separately mapped against family‐level COI biomarker sequences obtained through BOLD. Successfully mapped biomarker reads were then realigned and the resulting consensus sequences were extracted in Geneious Prime using MAAFT v7.490 (Katoh and Standley 2013).

Using biomarker gene consensus sequences generated for each sample, we constructed multiple sequence alignments using MAAFT v7.490, which contained previously published sequences of related taxa for phylogenetic comparisons. The generated alignments were provided as input for modeltest‐ng v0.1.7 (Darriba et al. 2020) to determine the best‐fit maximum likelihood model for generating phylogenies. We inferred maximum likelihood trees using RAxML (Stamatakis 2014) based on 1000 bootstrap iterations (see Supporting Information for information on parameters and taxa selected for outgroups). Estimates of genetic distance between clades were assessed using the Kimura two‐parameter (K2P) model of evolution calculated through MEGA v11.0.1. The best‐scoring trees and associated bipartition bootstrap frequencies were visualized using FigTree v1.4.4 and the Interactive Tree of Life (iToL).

## 3 Results

### 3.1 Performance of Field Sequencing Experiments

We extracted gDNA from nine small mammals and four blood‐feeding insects. The small mammals included six bats, two rodents, and one marsupial; insects included three culicine mosquitoes and a single phlebotomine sand fly. Using prepared gDNA libraries, we sequenced samples with NAS targeting mtDNA over seven separate sequencing experiments. Total sequencing output by sample yielded variable amounts of long‐read data, largely dependent on the weight of gDNA used for library preparation and flow cell performance throughout the run (Table S3). Through assessment of pore count quality checks prior to sequencing initiation, we noted that the MinION and Flongle flow cells used for sequencing experiments did not lose a considerable number of available nanopores during transit to each field site. We did note that total sequencing output appeared somewhat lower than expected for a few samples (e.g., TK217513, TK217562), possibly due to inefficient barcode and adapter ligation reactions during library preparation (Table S3). Of the four insects sequenced, raw read counts from individual samples ranged from 163,808 to 2,242,869, or between 75.8 and 1270.9 Mb (Table 1). Across each of the nine small mammals sequenced, read counts ranged between 30,666 and 405,269 total reads, amounting to between 20.9 and 185.8 Mb of total sequence data (Table 1). Raw reads were quality and length filtered with thresholds specified to remove poor quality reads, short reads, and any excessively long reads (i.e., non‐mitochondrial). Across all samples, the majority of reads survived these filtering steps. For small mammal samples, between 80.22% and 95.45% of reads successfully survived quality and read‐length filtering, while a lower proportion of reads, between 61.45% and 86.10%, were successfully retained across the four insect samples sequenced (Table 1).

**TABLE 1:** Field sequencing output and mtDNA reads isolated following quality score and read‐length filtering. Filtering was performed using a q‐score of 7 and above and with read lengths between 300 bp and 17 kb. Filtered reads were mapped to mitogenome references and isolated mtDNA reads were used as input for de novo mitogenome assembly and generation of biomarker gene consensus sequences.

Specimen ID	Group	Field site	Total bases (Mb)	Total reads	Total filtered reads	Reads surviving quality & length filtering	Filtered read N50	Total mapped mtDNA reads	Mitogenome assembled? (draft coverage)	Mitogenome length
**Small mammals**										
*Rattus rattus* TK217506	Rodent	MAH	50.6	62,025	49,755	80.22%	1057	1176	Yes (13×)	16,307
*Rattus rattus* TK217507	Rodent	MAH	119.3	138,362	114,917	83.06%	1071	2743	Yes (29×)	16,303
*Carollia* sp. TK217513	Bat	MAH	20.9	30,666	26,522	86.49%	718	1890	Yes (6×)	16,756
*Myotis c.f. simus* TK217523	Bat	MAH	114.3	165,248	157,725	95.45%	584	874	Yes (43×)	17,038
*Carollia* sp. TK217562	Bat	KWE	35.8	63,412	54,342	85.70%	590	1607	Yes (15×)	16,710
*Carollia* sp. TK217563	Bat	KWE	43.5	94,577	85,542	90.45%	475	658	Yes (49×)	16,764
*Trachops cirrhosus* TK217600	Bat	KWE	185.8	405,269	369,551	91.19%	477	1609	Yes (28×)	16,782
*Monodelphis brevicaudata* TK217608	Marsupial	KWE	182.8	392,148	362,509	92.44%	482	1844	Yes (45×)	17,060
*Pteronotus rubiginosus* TK217611	Bat	KWE	89.9	195,803	176,623	90.20%	476	1423	Yes (69×)	16,822
**Insects**										
*Mansonia titillans* CUL01	Culicine mosquito	MAH	1270.9	2,242,869	1,752,479	78.14%	764	14,927	Yes (126×)	16,675
*Mansonia titillans* CUL02	Culicine mosquito	MAH	579.8	1270,002	780,429	61.45%	641	6662	Partial	N/A
*Culex theobaldi* CUL06	Culicine mosquito	KWE	75.7	163,808	127,703	77.96%	535	1496	Partial	N/A
*Trichophoromyia ininii* PHL01	Phlebotomine sand fly	KWE	611.6	1,129,496	972,512	86.10%	564	9967	Yes (195×)	14,966

### 3.2 Mitochondrial Genome Assemblies

**FIGURE 3:**
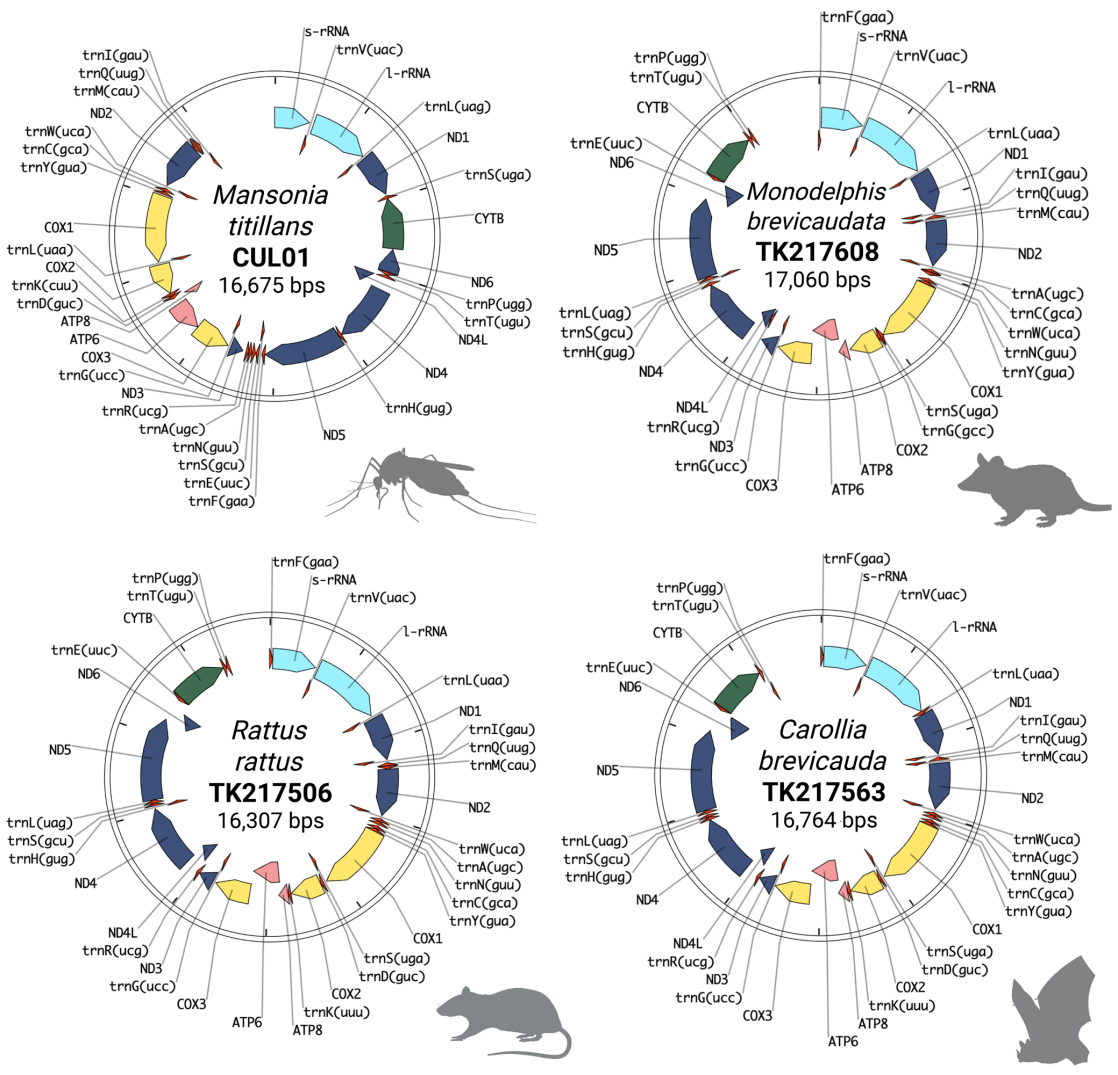
Representative mitochondrial genome maps depicting gene order and annotations for species from four taxa—a mosquito, *Mansonia titillans* (upper left), a marsupial, *Monodelphis brevicaudata* (upper right), a rodent, *Rattus rattus* (lower left), and a bat, *Carollia brevicauda* (lower right)—sequenced across both Guyana field sites using the mobile lab.

Filtered reads for each sample were queried against mitochondrial genome reference databases obtained through NCBI RefSeq using DNA‐ and protein‐based alignment strategies through the packages minimap2 and DIAMOND, respectively. The number of isolated mtDNA reads ranged widely by sample and as expected, largely correlated with the total number of reads obtained following filtering (Table 1 and Figure S1). Among insects sequenced, putative mtDNA read counts ranged between 1496 and 14,927, whereas for small mammals this ranged between 658 and 2743 mtDNA reads (Figure S2). Using these isolated putative mtDNA reads, complete circular mitochondrial genomes were assembled de novo, polished, and annotated for all nine small mammals; a culicine mosquito, CUL01; and the phlebotomine sand fly, PHL01 (Figure 3). The assembled mitogenomes were all consistent in terms of expected total length for their respective taxa. Small mammal mitogenomes ranged from 16,303 to 17,060 bp in length, while the CUL01 mosquito and PHL01 sand fly mitogenomes were assembled to a total length of 16,675 bp and 14,966 bp, respectively. Due to the variable amount of mtDNA reads used as input across all assemblies, the depth of mitogenome coverage varied markedly, ranging from roughly 6 to 195× in coverage (Table 1). Despite variations in coverage depth, all mitogenomes appeared assembled and annotated in full. Total mitogenome GC content was consistent with those reported for other small mammals and dipterans: small mammal GC% ranged from 34.1% to 42.1%, while the two insect mitogenome assemblies contained GC% ranging from 15.4% to 20.1% (Cameron 2014; Franco‐Sierra and Díaz‐Nieto 2020). We also compared gene content for each assembly and noted that our assemblies were complete in terms of the expected gene order across the mitogenome based on those reported for other small mammals and dipteran insects (Figure 3) (Boore 1999). Interestingly, we noted that de novo mitogenome assembly was unsuccessful in two of four insects sequenced: CUL02 and CUL06 (Table 1). In these samples, flye assemblies included an incomplete mitogenome assembled over multiple fragmented contigs. As an alternative approach for these two insect samples, we performed remapping and alignment of isolated mtDNA reads against BOLD Culicidae COI references and were successful at generating COI‐only consensuses for these samples for use in downstream phylogenies.

### 3.3 Phylogenetic Analysis and Species Identification

We next built multiple sequence alignments for systematic analyses using assembled biomarker consensus sequences obtained from annotated mitogenome assemblies or through alternative read remapping and alignment. Due to the availability of sequences from public repositories for comparisons, we elected to use COI gene sequences for insect phylogenies, and cytb gene sequences for small mammal phylogenies. After trimming alignments to phylogenetically informative lengths, modeltest‐ng was used to find the best fit model for nucleotide substitution, which was determined as GTRGAMMAIX (General Time Reversible + Gamma + Invariable Sites) for all alignments queried. We constructed a total of five phylogenetic trees using maximum likelihood analysis through RAxML, each built on 1000 bootstrap iterations. For small mammals, separate phylogenies were constructed for bats, rodents, marsupials based on the cytb gene. Construction of the bat phylogeny included the Old World flying fox *Pteropus dasymallus* (NC002612) as an outgroup, while outgroups for the rodent and marsupial phylogenies were specified as *Maxomys bartelsii* (KY54028) and *Perameles bougainville* (KJ868136), respectively. For insects, we built a culicid COI phylogeny for the three mosquitoes sequenced, with the anopheline mosquito *Anopheles crucians* (MT040804.0) serving as an outgroup; a phlebotomine COI phylogeny for the sand fly PHL01 was also built, with the Psychodidae drain fly *Clogmia albipunctata* (OK076900) as the outgroup.

The phylogenetic tree resulting from maximum likelihood analysis for bat cytb sequences allowed for confident molecular placement of all six specimens sequenced with the field lab. At the Mahaica field site, we evaluated two specimens: TK217513 and TK217523, identified via morphology as *Carollia* sp. and *Myotis c.f. albescens*, respectively. At the Kwebana site, we evaluated four bats: TK217562, TK217563, TK217600, and TK217611, identified morphologically in the field as *Carollia c.f. brevicauda*, *Carollia* sp., *Trachops cirrhosus* , and *Pteronotus parnellii* , respectively.

**FIGURE 4:**
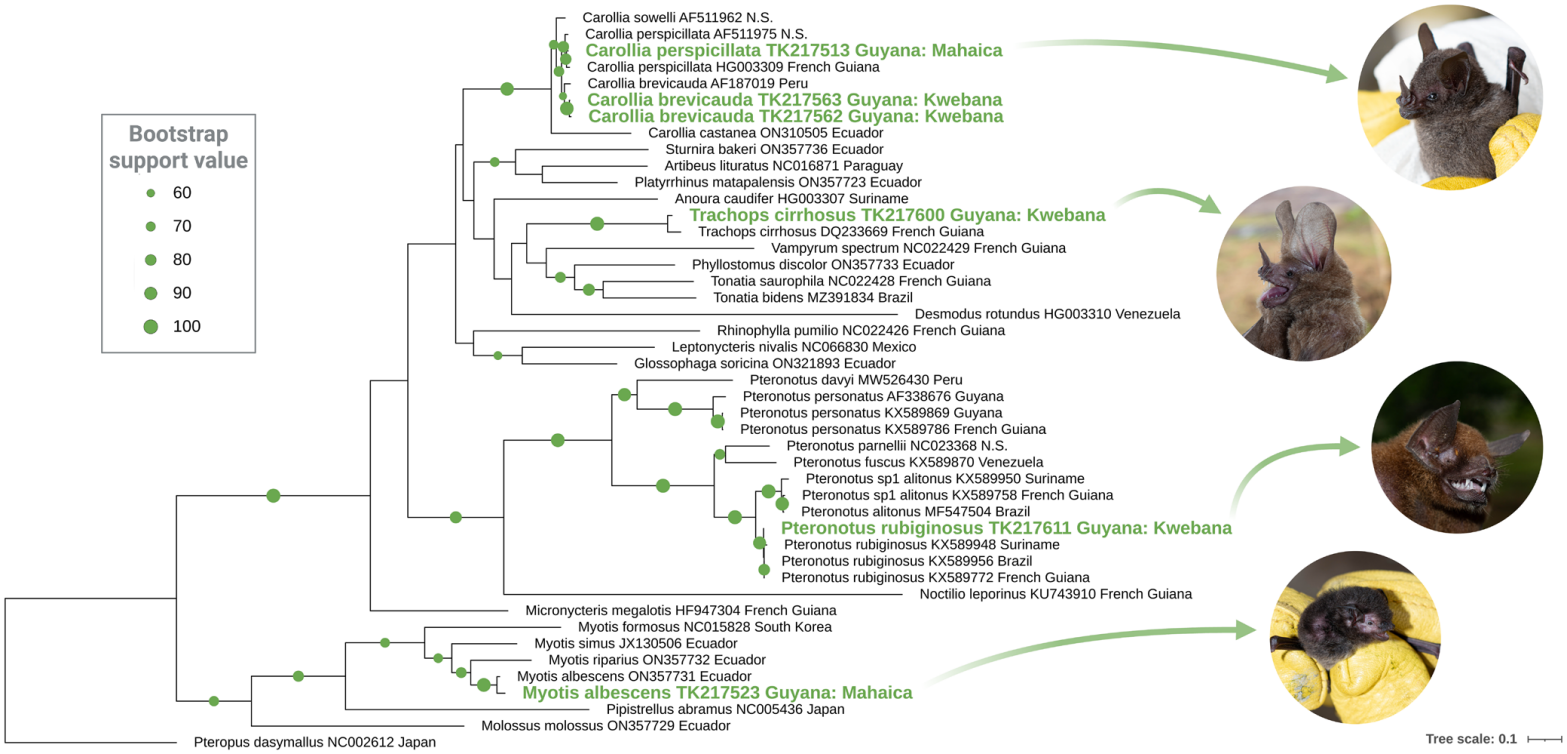
Phylogeny of bats inferred through maximum likelihood analysis based on *cytb* gene sequences. Consensus *cytb* sequences extracted from mitogenome assemblies generated using the mobile lab are highlighted in green. Bootstrap support values greater than 60 are scaled by size on corresponding nodes and shown as green circles. This tree was generated using 1000 bootstrap replicates, the GTRGAMMAIX models for nucleotide substitution and rate heterogeneity, and the taxon *Pteropus dasymallus* (NC002612) was selected for an outgroup. Bat photos by C.A.S.

Of the three *Carollia* specimens sequenced, the Mahaica individual (TK217513) was placed as *C. perspicillata* , while the Kwebana specimens (TK217562 and TK217563) were placed as the sister species, *C. brevicauda* (Figure 4). In the same tree, both the *Myotis* specimen (TK217523) and *Trachops* specimen (TK217600) were placed molecularly in clades with high statistical bootstrap support (i.e., 98–100 bootstrap support values) and identified in agreement with our morphologic findings as *M. albescens* and *T. cirrhosus* (Figure 4). Genetic distances, as estimated by K2P values, were calculated as ~2.67% for TK217523 and its nearest neighbor, *M. albescens* ON357731; and ~4.01% for TK217600 and *T. cirrhosus* DQ233669. Molecular identification of TK217611 was not in agreement with our morphologic ID of *P. parnellii* and instead supported its placement as *P. rubiginosus* in a clade with high bootstrap support and K2P genetic distances between *P. rubiginosus* sequences less than 2% (Figure 4).

We also constructed phylogenetic trees based on cyb gene consensus sequences generated for the two rodent specimens: TK217506 and TK217507. Both rodents were collected at the Mahaica field site and identified morphologically as *Rattus rattus*. Maximum likelihood analysis supported these morphologic findings, placing both cytb consensus sequences in a monophyletic clade with other *R. rattus* sequences with high bootstrap support throughout (Figure S3). Among the ~30 other *R. rattus* sequences included in this phylogeny, we additionally assessed whether we could observe clustering by sample country of collection (as listed in the accompanying NCBI metadata for each sequence) and observed no clear clustering patterns based on sample origin (Figure S3). For specimen TK217608, we constructed a marsupial‐focused phylogenetic tree, also based on cytb gene sequences. This specimen was collected at the Kwebana field site and identified morphologically as *Monodelphis brevicaudata* . Our resulting phylogeny supported this morphologic finding and depicted a monophyletic relationship for *M. brevicaudata* in a statistically well‐supported clade with K2P genetic distances of 0.4%–0.6% separating TK217608 from the two related *M. brevicaudata* cytb sequences (Figure S4).

Among the four insects assessed, maximum likelihood analyses enabled molecular species identifications with high confidence and supported our morphologic field identifications. In the culicid phylogenetic tree, mosquitoes CUL01 and CUL02—both sequenced at the Mahaica site and identified morphologically as *Mansonia* c.f. *titillans*—clustered with other *Ma. titillans* COI sequences in a monophyletic relationship and statistically supported clade (bootstrap value of 99) with limited genetic distance separating *Ma. titillans* sequences (Figure S5). The third mosquito, CUL06, from the Kwebana site—identified morphologically to genus level as *Culex* sp.—was identified molecularly as *Cx. theobaldi* based on its clustering with two other *Cx. theobaldi* sequences in a monophyletic clade, also with strong statistical support and minimal genetic distance (bootstrap value of 100) (Figure S5). The maximum likelihood analysis for the sand fly, PHL01, suggested its molecular identification as *Trichophoromyia ininii*, as our COI consensus clustered with *T. ininii* in a statistically supported clade and with roughly 2.16% K2P genetic distance separating our PHL01 sequence and its closest neighbor, *T. ininii* KX356040 (Figure S6).

## 4 Discussion

Incorporation of molecular systematic data alongside morphologic information (i.e., integrative taxonomy) has considerably advanced animal species identification efforts, particularly among cryptic and non‐field distinguishable groups (Franco‐Sierra and Díaz‐Nieto 2020; Hebert, Ratnasingham, et al. 2003; Pante et al. 2015). Here, we describe how a mobile lab outfitted for long‐read ONT sequencing and targeted enrichment through NAS is effective at rapid generation of in situ molecular systematic data for phylogenetic analysis and molecular species identification. For field analyses, we strategically selected for a wide range of animal taxa notoriously difficult to identify based on morphology alone (Gardner 2019; Lane 1953; López‐Baucells et al. 2018). Across both field sites, we rapidly generated mitochondrial biomarker consensus sequences to enable amplification‐free identification of six bats of multiple genera (*Carollia*, *Myotis*, *Trachops*, *Pteronotus*), rodents of the genus *Rattus*, and a *Monodelphis* marsupial, three culicine mosquitoes, and a phlebotomine sand fly.

There is a fast‐growing body of literature focused on the use of portable molecular technologies and their applications toward real‐time, on‐the‐ground genomics (Chen et al. 2023; Urban et al. 2023; Watsa et al. 2020). While previous studies have similarly employed mobile labs with ONT sequencing for species barcoding across diverse field locations, earlier efforts have been largely focused on PCR‐based approaches (Blanco et al. 2020; Chang et al. 2020; Latorre‐Pérez et al. 2020; Pomerantz et al. 2018; Urban et al. 2021). While PCR is a viable option in field settings, its use in mobile labs is accompanied by several weaknesses. Many of the currently available options for miniaturized PCR thermocyclers (e.g., MiniPCR, Biomeme, BentoLab) have heavily constrained throughput, significantly limiting the number of samples that can be screened at once (Chang et al. 2020; Russell et al. 2018). Moreover, optimization of PCR assays for the field can be a problematic and time‐consuming process, with even well‐established assays prone to failure. In their work on reptile and amphibian species barcoding in the Ecuadorian rainforest, Pomerantz et al. noted that despite observed PCR amplification in their 16S and ND4 mitochondrial gene PCR assays, the vertebrate cytb assay in their mobile lab failed for reasons that were difficult to troubleshoot in the field (Pomerantz et al. 2018).

As a way to bypass reliance on amplification‐based methods, we elected to avoid PCR entirely during our mobile lab deployment and instead used NAS to target and enrich for mtDNA reads across the mitochondrial genome. NAS serves as the only described method for targeted sequencing wherein sequence enrichment is achieved solely through software‐based bioinformatic means, reducing the need for incorporation of additional steps or reagents while samples are being prepared for sequencing (Payne et al. 2021). Previous work has demonstrated that NAS can be used to effectively sequence and assemble novel animal mitochondrial genomes using mitogenomes of related species as enrichment targets (Frank et al. 2024; Kipp et al. 2023; Wanner et al. 2021). Our findings here offer added support in the broad utility of NAS for mitochondrial genomics through ‘phylogenetic capture’ methods, as our field sequencing experiments were successful at capturing considerable amounts of long‐read mtDNA data, including for species lacking published mitogenomes (i.e., *Ma. titillans*, *Cx. theobaldi*, *M. brevicaudata* ) (Table 1 and Figure S1).

As highlighted above, bats across many genera, such as *Myotis*, share morphologic characteristics and largely necessitate molecular data for accurate species differentiation, especially in regions of sympatry (Novaes et al. 2024). *Myotis* species are distributed globally and diversity in the Neotropics is especially high (Carrión Bonilla et al. 2024; R. J. Larsen et al. 2012). Due to their widespread cryptic variation, species diversity of South American *Myotis* is likely underestimated, with numerous lineages likely warranting species‐level status (Carrión Bonilla et al. 2024; R. J. Larsen et al. 2012; Novaes et al. 2024). In our field experiments, we examined a *Myotis* specimen (TK217523) and confirmed the specimen as *M. albescens* within 48 h of its collection (Figure 4). Likewise, bats of the genera *Pteronotus* and *Carollia* are also highly difficult to morphologically differentiate from conspecifics, often requiring phylogenetic data to elucidate species boundaries (Benítez et al. 2021; Dávalos 2006; Hoffmann and Baker 2003; Pavan et al. 2018; Pavan and Marroig 2016). From a taxonomic perspective, both *Pteronotus* and *Carollia* have undergone extensive revision over the past few decades, and, in the absence of up‐to‐date phylogenetic data, accurate species identifications are particularly challenging (Pavan et al. 2011; Thoisy et al. 2014). In Guyana, several species of *Pteronotus* (e.g., *P*. *altonius*, *P. parnellii* , *P. rubiginosus* ) and *Carollia* (e.g., *C. brevicauda* , *C. perspicillata* , *C. castanea* ) occur sympatrically with congeners, serving to confound field‐based identifications (Dávalos 2006; Pavan and Marroig 2016). As our findings demonstrate, in situ phylogenetic analyses can provide incredible opportunities for researchers aiming to accurately catalog the biodiversity of these broadly distributed and relatively common cryptic species. Our results here document *P. rubiginosus* in western Guyana as well as two closely related sister species of *Carollia*: *C. perspicillata* and *C. brevicauda* , with *C. perspicillata* collected in our eastern Mahaica field site and *C. brevicauda* in our western Kwebana field site.

Similarly, accurate species identification across mosquitoes, sand flies, and other hematophagous insects can be particularly difficult using morphology alone, owing to widespread cryptic variation and conserved morphologic features. A considerable amount of taxonomic expertise is frequently required to arrive at species rank identifications morphologically, especially in regions of the world where species diversity in hematophagous insect communities is less well studied (Alquezar et al. 2010; Beebe 2018; Chan‐Chable et al. 2019; Contreras Gutiérrez et al. 2014; X. Li and Wiens 2023; Young and Duncan 1994). Moreover, taxonomic keys for hematophagous insects in certain regions may be outdated, unable to differentiate select species, or not fully encompassing true species diversity (Forattini 2002; Lane 1953; Young and Duncan 1994). Complicating morphology‐based identification efforts further is the observation that many commonly used methods for collecting these blood‐feeding insects (e.g., CDC light traps) lead to inadvertent destruction of wings, scales, and other identifying features (Beebe 2018). To avoid reliance on these techniques, we utilized our mobile lab and mtDNA sequencing approaches to rapidly identify three mosquito specimens belonging to two species: *Mansonia titillans* and *Culex theobaldi. Ma. titillans* is widely distributed and often locally abundant culicid throughout South America and is a generalist feeder of birds and mammals, including humans (Dos Santos Silva et al. 2012; Stein et al. 2013). Across its wide range, its role in arbovirus transmission has not been well characterized, though few individuals of the species have been found naturally infected with Venezuelan equine encephalitis virus and St. Louis encephalitis virus (Beranek et al. 2018; Stein et al. 2013). *Cx. theobaldi* has been recorded across Mexico, Central America, and South America, though its presence in field collections is often at low abundance; as a result, notably little is known about its ecology and status as a potential vector species (Ortega‐Morales et al. 2018). We additionally sequenced a singly phlebotomine sand fly specimen from the Kwebana site, which was identified through systematic analysis as *Trichophoromyia ininii*. Our rapid, field‐based molecular identification here is notable, as morphologic identification in sand flies is traditionally a laborious process involving the mounting of specimens on slides and visualization under a compound microscope to view fine‐scale features of the head and genitalia (Contreras Gutiérrez et al. 2014; Young and Duncan 1994). Reports of *T. ininii* are infrequent, though it has been collected from regions bordering Guyana in Brazil and elsewhere in the Guianan Shield; though the species appears to prefer forested settings, it has been reported from peridomestic environments and has also been found to be naturally infected with the parasite *Leishmania braziliensis*, capable of causing mucocutaneous leishmaniasis in humans (dos Santos and Silveira 2020; Vasconcelos dos Santos et al. 2019).

Although our collective findings demonstrate the wide‐ranging potential of NAS‐based approaches for in situ species molecular systematics and species barcoding, there are important limitations of note and we did encounter several challenges during this study. A key limitation is that all data generated during our field sequencing efforts utilized the NAS strategy and was not accompanied by control sequencing experiments or partitioning of flow cells to capture both non‐targeted and enriched NAS data. As such, our findings here suggest the feasibility of NAS as an effective method for capturing mitochondrial reads to enable real‐time systematics; however, future studies are warranted to further evaluate NAS and its efficacy at enriching for mitogenomes or other phylogenetically informative targets in both lab and field settings. In addition, the total sequence data output achieved for some specimens (e.g., TK217513, TK217562) was lower than anticipated. Across all samples, we noted that data output was somewhat reduced in comparison to that of typical ONT sequencing experiments by our group in our established BSL‐2 molecular lab. We believe that this was due to inefficient adapter ligation reactions, as pore occupancy observed during field sequencing runs was lower than expected (< 80%). It is likely that these ligation reactions during library preparation exhibited reduced efficiency, as temperatures during sample preparation were often in excess of the optimal ~25°C for reaction incubation. Additionally, while many ONT library preparation reagents are stable at ambient temperatures, ligase enzymes used for barcode and adapter ligation reactions are particularly vulnerable to degradation if not stored frozen between uses; our reliance on intermittent generator‐based electricity to keep reagents cold may have led to freezing–thawing of samples and caused some damage to ligase enzymes limiting their efficiency. Ultimately, however, the use of NAS may have helped to some degree to overcome these limitations in throughput, as a sufficient proportion of the long‐read data that were generated belonged to mtDNA targets (Table 1). We were able to generate complete mitogenomes de novo for most specimens sequenced; however, we did note that assembly was unsuccessful in two mosquito specimens. Interestingly, the number of mtDNA reads obtained for these two mosquito specimens (CUL02 and CUL06) was in excess of that obtained by multiple small mammal specimens for which mitogenome assemblies were successful (Table 1 and Figure S1). BLAST analyses performed on a subset of these CUL02 and CUL06 reads suggested that some contained nuclear mtDNA segments (NUMTs), which were inadvertently captured through NAS. Mitochondrial pseudogenes present in NUMTs can frequently confound insect mitogenomic studies (Hlaing et al. 2009; Hebert et al. 2022). Although their presence in our mtDNA data sets may have precluded our ability to generate de novo mitogenome assemblies for these two samples, we were nevertheless able to generate COI consensus sequences through our alternative read remapping and alignment steps. In addition to the limitations above, we encountered numerous bioinformatic challenges in the field, as many of the software packages used for local data processing and analysis were run interactively on our local computer or through an assortment of bash scripts. The amount of time spent by our group processing NAS data in the field could have been greatly reduced by improved software automation and development of NAS‐specific bioinformatic pipelines. Broadly speaking, implementation of bioinformatic tools is likely to be a significant challenge across many groups seeking to build and deploy their own mobile labs. While some software packages have been developed focused on building consensus biomarker sequences from lab‐ and field‐generated nanopore amplicon data (Krehenwinkel et al. 2019; Pomerantz et al. 2022; Sahlin et al. 2021; Vierstraete and Braeckman 2022), end‐to‐end bioinformatic pipelines designed for targeted NAS‐derived data and whole mitogenome‐based approaches are in need of development in future work.

As animal species continue to adapt to rapidly changing environments, targeted and field‐focused methods such as these will be critical toward documenting shifting ranges and facilitating real‐time species barcoding. Accurate molecular assessments of animal species through barcoding are an essential part of making informed and timely decisions on species conservation efforts and mitigating the risk of emerging vector‐borne and zoonotic diseases. In this context, field‐based approaches that incorporate rapid generation of molecular data for systematic analysis are increasingly valuable, particularly in highly biodiverse tropical environments where previous collection records across many faunal groups are not well represented. Our results here document NAS as a versatile tool for use in the field and for incorporation into portable molecular laboratories. While future studies are needed to further evaluate the field capabilities of NAS, we demonstrate that its use enabled us to streamline field laboratory workflows, moving quickly from extracted genomic DNAs to prepared sequencing libraries, while avoiding time‐consuming and potentially problematic PCRs. This approach enabled the generation of robust systematic data that could be processed bioinformatically in a rapid fashion and with relative ease, allowing us to taxonomically place samples obtained from a wide diversity of animal specimens soon after their collection. Collectively, these findings contribute to a growing body of research on the utility of mobile labs and their potential for building sequencing capacity across remote settings and settings with limited laboratory infrastructure. As portable genomic technologies continue to advance and researchers embrace mobile laboratories for field sequencing, the amplification‐free NAS strategies described here can help accelerate a wide range of initiatives aimed at characterizing species biodiversity, identifying cryptic animal taxa, and performing on‐the‐ground, real‐time biosurveillance of consequential pathogen vectors and hosts.

## Author Contributions

**Evan J. Kipp:** conceptualization (supporting), data curation (lead), formal analysis (lead), investigation (equal), methodology (equal), writing – original draft (lead), writing – review and editing (supporting). **Marissa S. Milstein:** conceptualization (supporting), data curation (supporting), formal analysis (supporting), funding acquisition (supporting), investigation (equal), methodology (supporting), project administration (lead), writing – original draft (supporting), writing – review and editing (supporting). **Lexi E. Frank:** conceptualization (supporting), data curation (supporting), formal analysis (supporting), investigation (equal), methodology (supporting), writing – review and editing (supporting). **Roxanne J. Larsen:** investigation (equal), methodology (supporting), writing – review and editing (supporting). **Tiffany M. Wolf:** funding acquisition (equal), investigation (equal), methodology (supporting), writing – review and editing (supporting). **Christopher Faulk:** conceptualization (supporting), methodology (supporting), writing – review and editing (supporting). **Christopher A. Shaffer:** funding acquisition (supporting), investigation (equal), methodology (supporting), project administration (lead), writing – review and editing (supporting). **Peter A. Larsen:** conceptualization (lead), data curation (supporting), formal analysis (supporting), funding acquisition (equal), investigation (equal), methodology (equal), writing – original draft (equal), writing – review and editing (supporting).

## Conflicts of Interest

The authors declare no conflicts of interest.

## Supporting information


**Appendix S1:** ece372442‐sup‐0001‐AppendixS1.docx.

## Data Availability

Raw nanopore sequencing data, NAS enrichment references, and multiple sequence alignment files generated during this project are deposited on Dryad (https://doi.org/10.5061/dryad.bnzs7h4p2). Consensus barcoding gene sequences used for phylogenies are deposited on NCBI GenBank under accession numbers PX273940–PX273948 (small mammals) and PX262133–PX262136 (insects). The code and shell commands used to perform the analyses are available in the Supporting Information.
