# Supplementary material for: Going Mobile: Using Portable Genomic Technologies for PCR‐Free In Situ Species Identification and Real‐Time Molecular Systematics
Source: Ecol Evol. 2025 Nov 5;15(11):e72442. doi: 10.1002/ece3.72442 (PMC12588770; doi:10.1002/ece3.72442)
Supplement: Supplementary file 1 — Appendix S1: ece372442‐sup‐0001‐AppendixS1.docx. [file ECE3-15-e72442-s001.docx]

**Supporting Information for:**

**Going mobile: using portable genomic technologies for PCR-free *in situ* species identification and real-time molecular systematics**

Evan J. Kipp, Marissa S. Milstein, Lexi E. Frank, Roxanne J. Larsen, Tiffany M. Wolf, Christopher Faulk, Christopher A. Shaffer, Peter A. Larsen

| **Table S1.** | **List and costs of portable lab equipment** | Page 2 – 3 |
| --- | --- | --- |
| **Table S2.** | **Reagents and consumables** | Page 4 – 5 |
| **Table S3.** | **Raw sequencing output metrics** | Page 6 |
| **Figure S1.** | **Putative mtDNA read correlation plot** | Page 7 |
| **Figure S2.** | **Violin plot of putative mtDNA reads by length** | Page 8 |
| **Figure S3.** | **Phylogeny of *Rattus*** | Page 9 |
| **Figure S4.** | **Phylogeny of *Monodelphis*** | Page 10 |
| **Figure S5.** | **Phylogeny of culicine mosquitoes** | Page 11 |
| **Figure S6.** | **Phylogeny of phlebotomine sand flies** | Page 12 |

Table S1. List of portable lab equipment used during field sequencing experiments and estimates for costs of individual instruments. List excludes some conventional laboratory supplies used, such as micropipettes, tips, tubes, racks, and personal protective equipment (e.g., gloves, disinfectant, masks). Cost estimates provided are based at the time of purchasing and may not reflect current prices for each instrument.

| **Portable Lab Instruments** | | | | |
| --- | --- | --- | --- | --- |
| **Item** | **Manufacturer** | **Catalog Number** | **Purpose** | **Estimated Cost (USD)** |
| MinION Mk1b sequencer | ONT | MIN-101B | Sequencing nanopore libraries (MIN or FLG) | $1,000 |
| MiniPCR mini16 thermocycler | MiniPCR | QP-1016-16 | End-prep and DNA repair reactions; supplemental heat block | $860 |
| Gyro Plus Microcentrifuge, variable speed | MiniPCR | QP-1800-02 | DNA extractions with spin columns; spinning down tubes | $265 |
| BlueGel electrophoresis system with transilluminator | MiniPCR | QP-1500-01 | DNA extract visualization | $309 |
| Magnetic separation rack (6 tube) | NEB | S1506S | Bead cleanup steps; library prep | $231 |
| myBlock mini dry bath with 1.5 ml tube heat blocks | Benchmark | Multiple vendors | End-prep and DNA repair reactions; 37ºC elution steps | $350 |
| TerraLyzer Cell Disruptor | Zymo | N/A | Bead-beating, tissue lysis prior to DNA extraction | $1,221 |
| Mini vortex mixer | Fisher | N/A | Reagent preparation | $200 |
| Qubit 4 fluorometer | Invitrogen | Q33238 | Assessing DNA yield post-extraction and during library preparation | $3,900 |
| Stereomicroscope | Fisher | N/A | Insect sorting and morphologic identification | $322 |
| Linux laptop with RTX 3080 GPU | Dell | N/A | Primary sequencing computer with GPU for basecalling, analyses | $2,900 |
| Extreme Portable SSD (4TB) | SanDisk | Multiple vendors | Data backup; storing BLAST and kraken2 databases | $479 |
| USB computer fans 120 mm(2) | Multiple manufacturers through Amazon | N/A | Air circulation for MinION and sequencing laptop | $15 |
| High Performance Hard Sided Cooler (77 Quart) | Lifetime | Multiple vendors | Filled with ice packs and used for sample transport and storage | $174 |
| Greenhouse 'pop-up' tent | Multiple manufacturers through Amazon | N/A | Clean work environment for extractions and library preparation | $20 |
| **Total instrument and hardware costs:** | | | | $12,246 |

Table S2. List of consumable supplies used in the field and estimates of cost per unit and cost to perform targeted mtDNA sequencing for an individual sample using either a single-sample Flongle flow cell or MinION flow cell with barcoding capability. Cost estimates provided are based at the time of purchasing and may not reflect current prices for each consumable.

| **Consumables and Reagents for Sample Preparation** | | | | | | |
| --- | --- | --- | --- | --- | --- | --- |
| **Item** | **Manufacturer** | **Catalog Number** | **Purpose** | **Estimated Cost (USD)** | **Cost per sample (MinION)** | **Cost per sample (Flongle)** |
| Sequencing ligation kit | ONT | SQK-LSK109 | Primary kit for nanopore library preparation | $599 | $25 | $25 |
| Native barcoding kit | ONT | EXP-NBD104 | Preparation of multiplexed libraries | $188 | $1 | $1 |
| Axygen AxyPrep Mag PCR Clean-Up Kit (5mL) | Corning | MAG-PCR-CL-5 | Bead cleanup steps used throughout library prep | $140 | $3 | $3 |
| NEBNext FFPE DNA Repair Mix | NEB | M6630S | Initial end-prep and DNA repair reaction | $144 | $4 | $4 |
| NEBNext Ultra II End Repair/dA- Tailing Module | NEB | E7546S | Initial end-prep and DNA repair reaction | $212 | $5 | $5 |
| NEB Blunt/TA Ligase Master Mix | NEB | M0367S | Ligation of ONT barcodes | $111 | $4 | $4 |
| NEBNext Quick Ligation Module | NEB | E6056S | Adapter ligation | $278 | $14 | $14 |
| DNA/RNA Shield | Zymo | R1100-50 | Nucleic acid preservation solution | $68 | $1 | $1 |
| DNeasy Blood & Tissue Kit (50 extractions) | Qiagen | 69504 | Spin column extraction kit for genomic DNA isolation | $164 | $3 | $3 |
| Lo Bind Tubes, pack of 250 | Eppendorf | 22431021 | Microcentrifuge tubes used for library preparation | $43 | $1 | $1 |
| PowerBead Tubes Ceramic 2.8mm (50) | Qiagen | 13114-50 | Tissue disruption for mammal tissues | $165.00 | $3 | $3 |
| RNase-Free Disposable Pellet Pestles | Fisher | 12-141-364 | Tissue disruption for arthropods | $87 | $1 | $1 |
| Flow cell (R9.4.1) | ONT | FLO-MIN106D | Sequencing prepared (multiplexed) libraries | $900 | $75 | N/A |
| Flongle flow cell | ONT | FLO-FLG001 | Sequencing in combination with Flongle adapter | $90 | N/A | $90 |
| Flow Cell Priming Kit | ONT | EXP-FLP002 | Preparing flow cells for sequencing | $35 | $4 | $4 |
| Flow cell wash kit | ONT | EXP-WSH004 | Washing flow cells for re-use | $99 | $12 | $12 |
| **Total consumable costs per sample sequenced:** | | | |  | $157 | $172 |

Table S3. Raw sequencing summary statistics for small mammal and insects sequenced across both Guyana field sites.

| **Morphologic Field ID** | **Sample ID** | **Field Site** | **Flow Cell Type** | **Total Bases (Mb)** | **Total Reads** | **Raw Read N50** | **Mean Read Length** | **Mean Read Quality** |
| --- | --- | --- | --- | --- | --- | --- | --- | --- |
| **Small Mammals** | | | | | | | | |
| *Rattus rattus* | TK217506 | Mahaica | MinION | 50.6 | 62025 | 1038 | 816.4 | 13 |
| *Rattus rattus* | TK217507 | Mahaica | MinION | 119.2 | 138362 | 1057 | 862.2 | 13 |
| *Carollia sp.* | TK217513 | Mahaica | MinION (2nd use) | 20.8 | 30666 | 711 | 680.3 | 12.3 |
| *Myotis c.f. simus* | TK217523 | Mahaica | MinION | 114.2 | 165248 | 596 | 691.4 | 14.6 |
| *Carollia sp.* | TK217562 | Kwebana | Flongle | 35.8 | 63412 | 587 | 564.6 | 10.6 |
| *Carollia sp.* | TK217563 | Kwebana | MinION | 43.5 | 94577 | 470 | 460.3 | 12.9 |
| *Trachops cirrhosus* | TK217600 | Kwebana | MinION | 185.8 | 405269 | 474 | 458.5 | 12.6 |
| *Monodelphis brevicaudata* | TK217608 | Kwebana | MinION | 182.7 | 392148 | 480 | 466.1 | 12.8 |
| *Pteronotus parnelli* | TK217611 | Kwebana | MinION | 89.8 | 195803 | 472 | 459 | 12.7 |
| **Insects** | | | | | | | | |
| *Mansonia* sp. | CUL01 | Mahaica | MinION | 1270.9 | 2242869 | 706 | 566.6 | 12.5 |
| *Mansonia* sp. | CUL02 | Mahaica | MinION | 579.7 | 1270002 | 528 | 456.5 | 12.7 |
| *Lutzomyia* sp. | PHL01 | Kwebana | MinION (2nd use) | 611.6 | 1129496 | 561 | 541.5 | 11.3 |
| *Culex* sp. | CUL06 | Kwebana | MinION (2nd use) | 75.7 | 163808 | 514 | 462.4 | 12.2 |


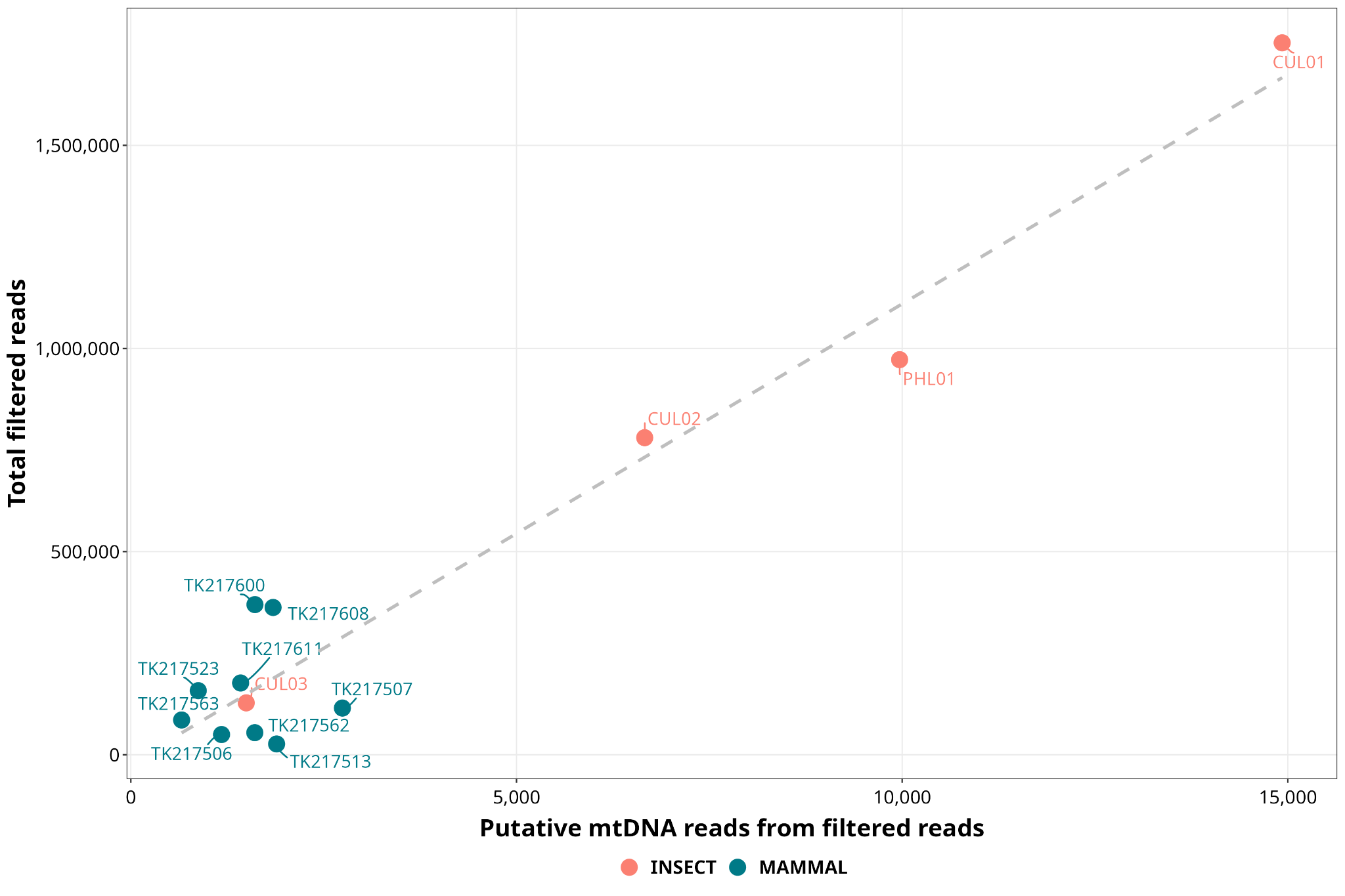


Figure S1. Correlation between total filtered nanopore reads and putative mtDNA reads obtained with NAS for small mammal and insect samples sequenced. The total number of filtered reads (y-axis) is plotted against the total number of putative mtDNA reads following DNA- and protein-based alignment (x-axis).


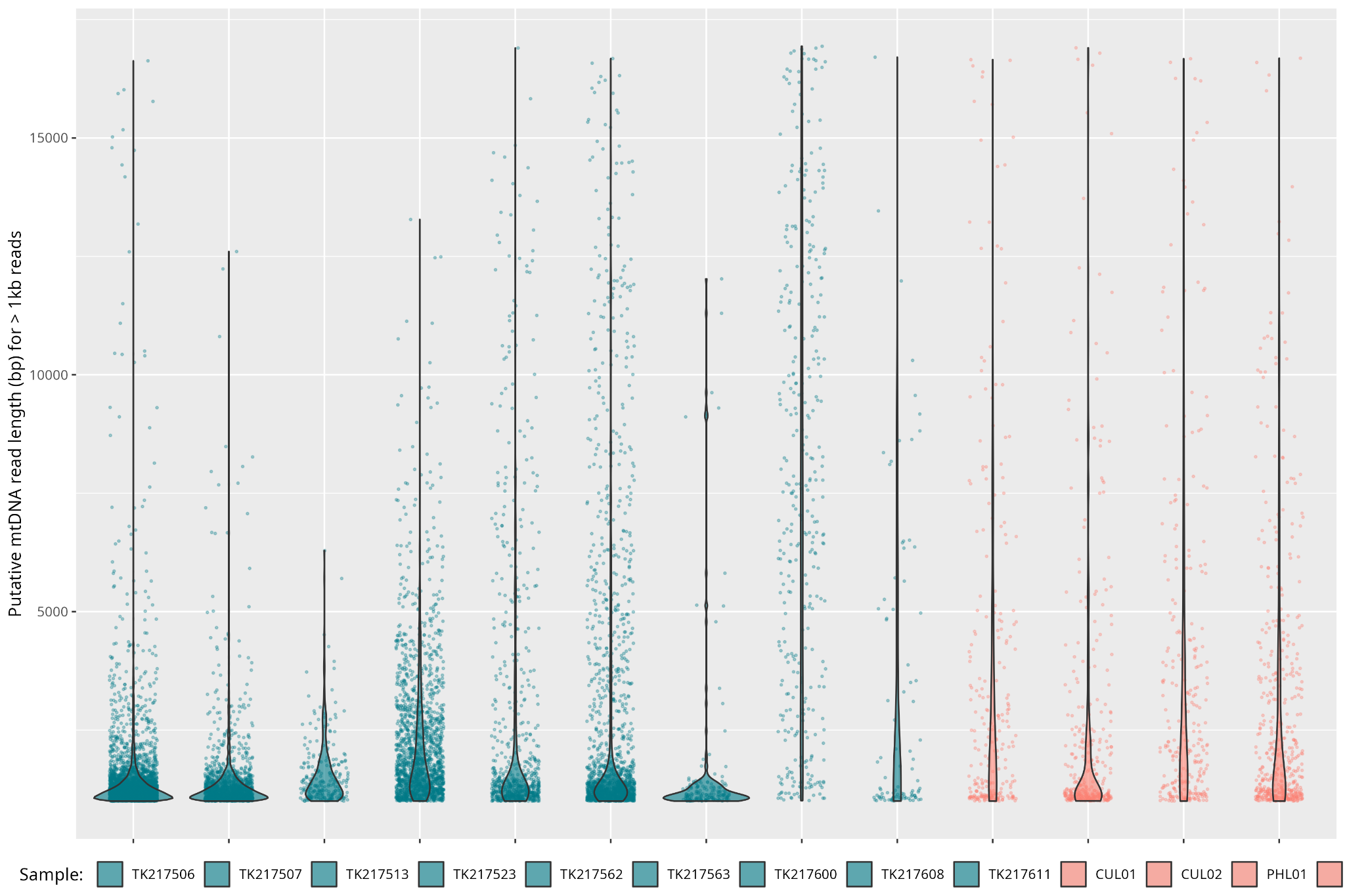


Figure S2. Violin plot of total putative mtDNA reads, plotted by read length, isolated following read quality and length filtering and used as input for mtDNA read recruitment prior to *de novo* mitogenome assemblies.


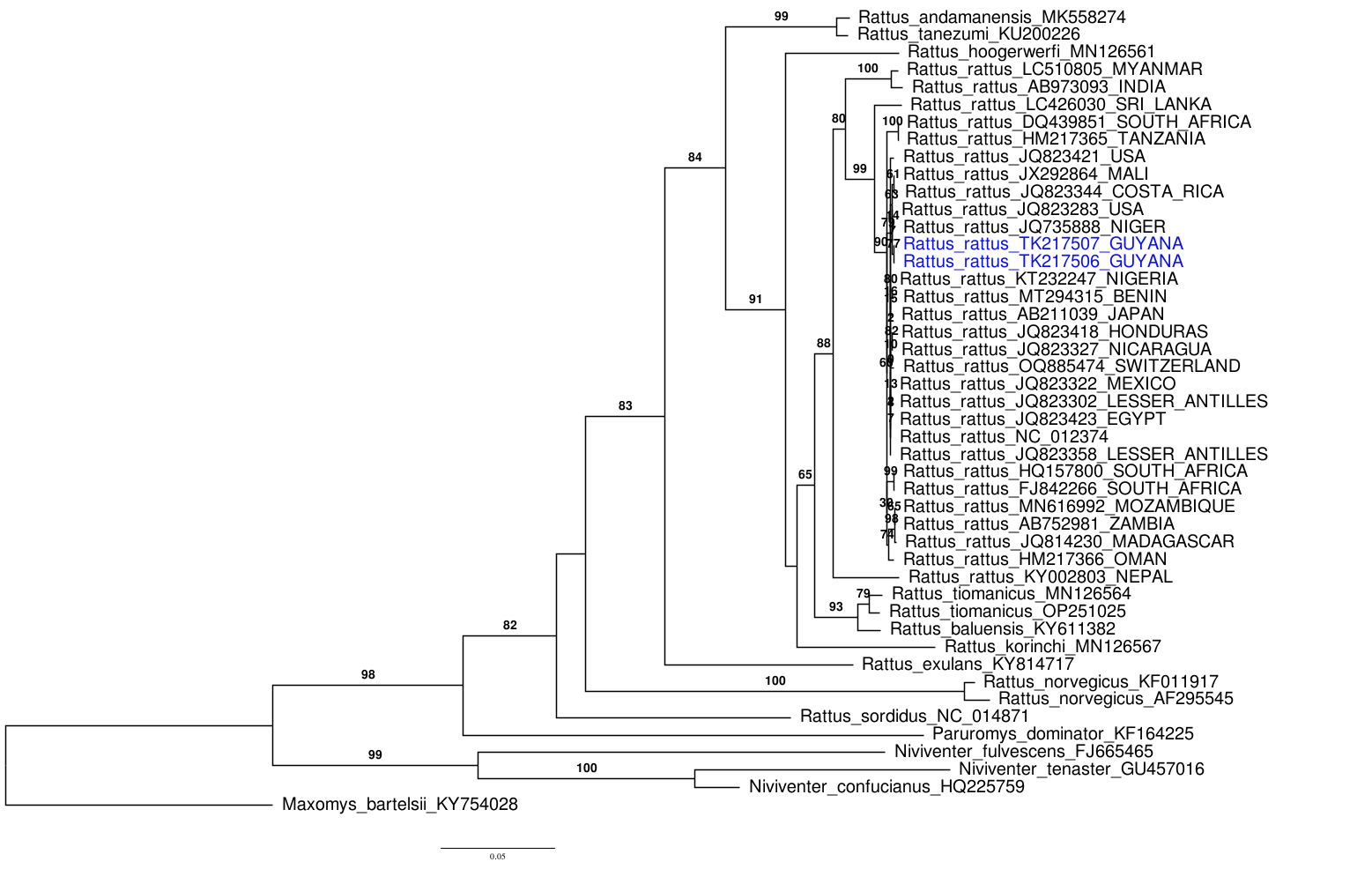


Figure S3. Maximum likelihood phylogeny of two *R. rattus* specimens collected and sequenced at the Mahaica field site in Guyana (labeled in blue). Tree was inferred using *cytb* gene sequences and based on 1,000 bootstrap replicates. Nodes with bootstrap support values greater than 60 are listed. Label identifiers for comparative sequences (black labels) represent NCBI Genbank accession numbers and, where applicable, are followed by country of sample origin as denoted in Genbank.


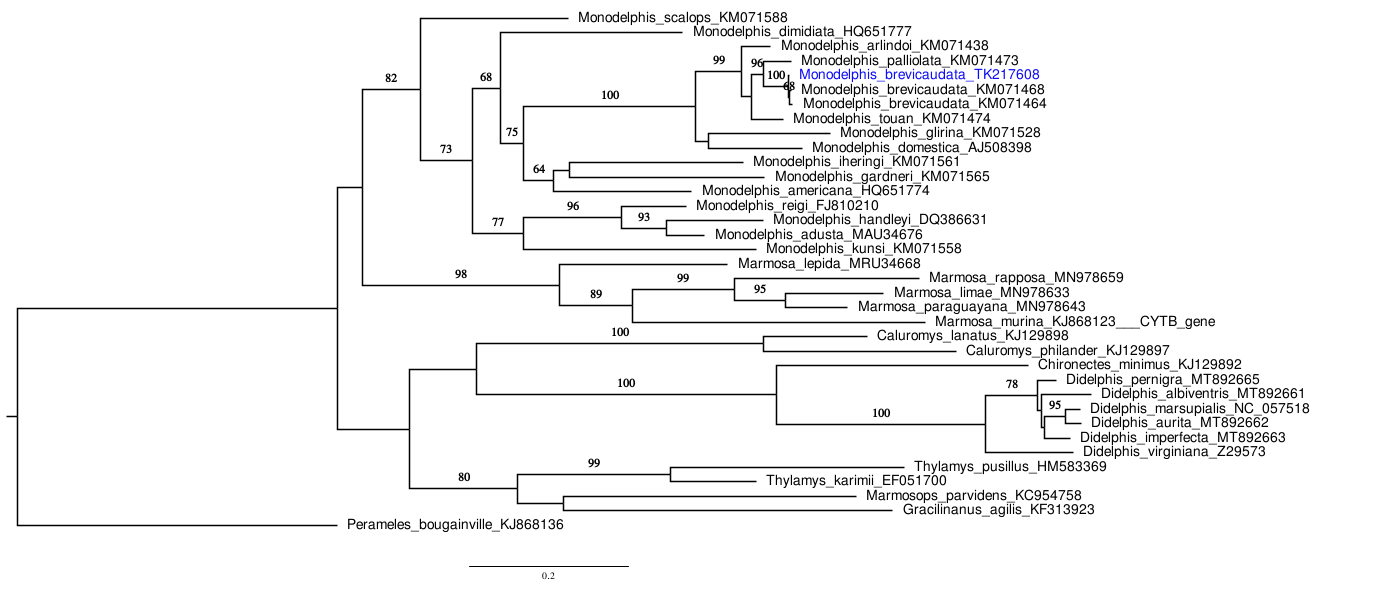


Figure S4. Maximum likelihood phylogeny of *M. brevicaudata* collected and sequenced at the Kwebana field site (labeled in blue). Tree was inferred using *cytb* gene sequences and based on 1,000 bootstrap replicates. Nodes with bootstrap support values of greater than 60 are listed. Label identifiers for comparative sequences (black labels) represent NCBI Genbank accession numbers.


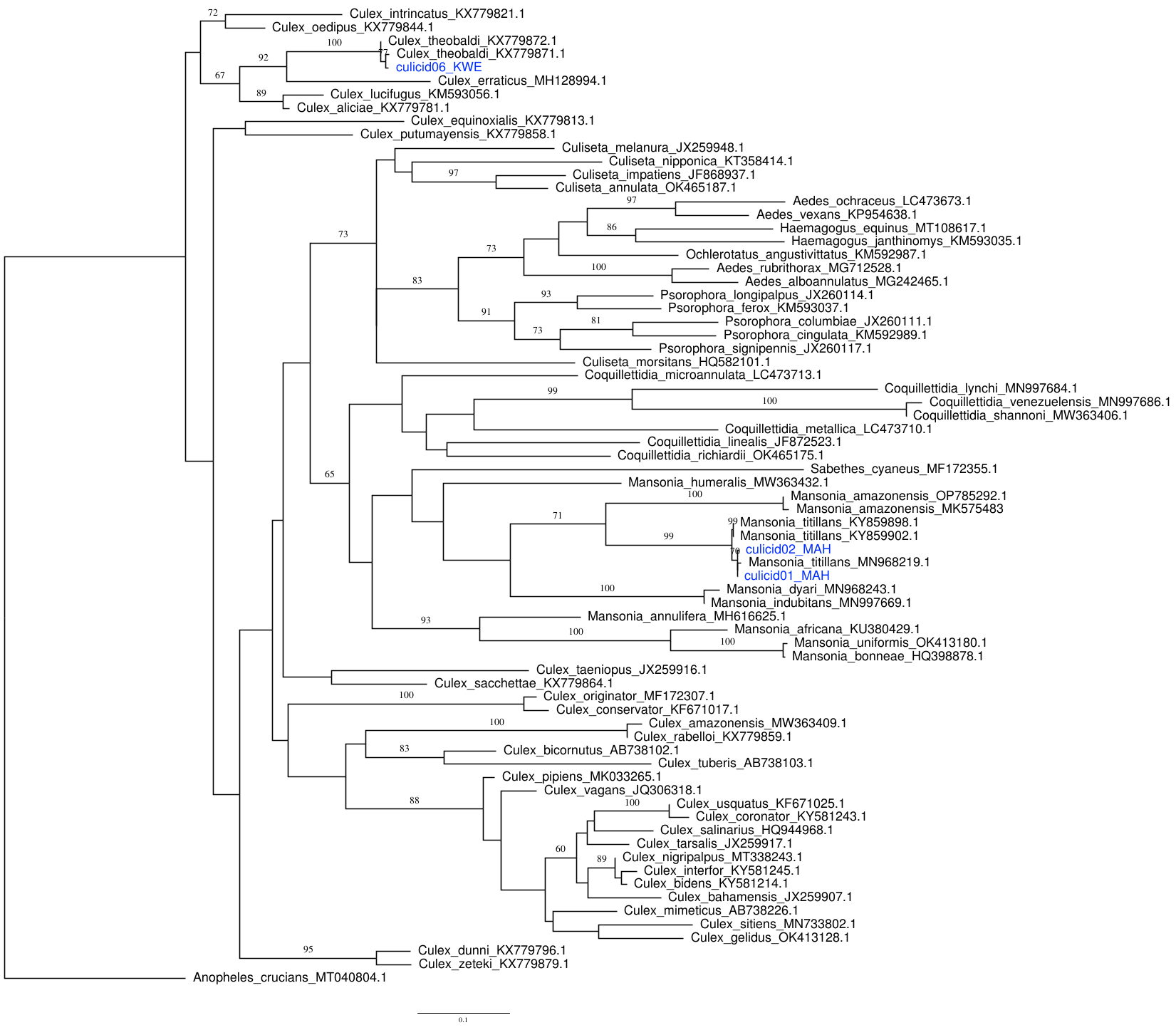


Figure S5. Maximum likelihood phylogeny of three culicid mosquitoes collected and sequenced across both Guyana field sites (labeled in blue). Tree was inferred using *COI* gene consensus sequences and based on 1,000 bootstrap replicates. Nodes with bootstrap support values of greater than 60 are listed. Label identifiers for comparative sequences (black labels) represent NCBI Genbank accession numbers.


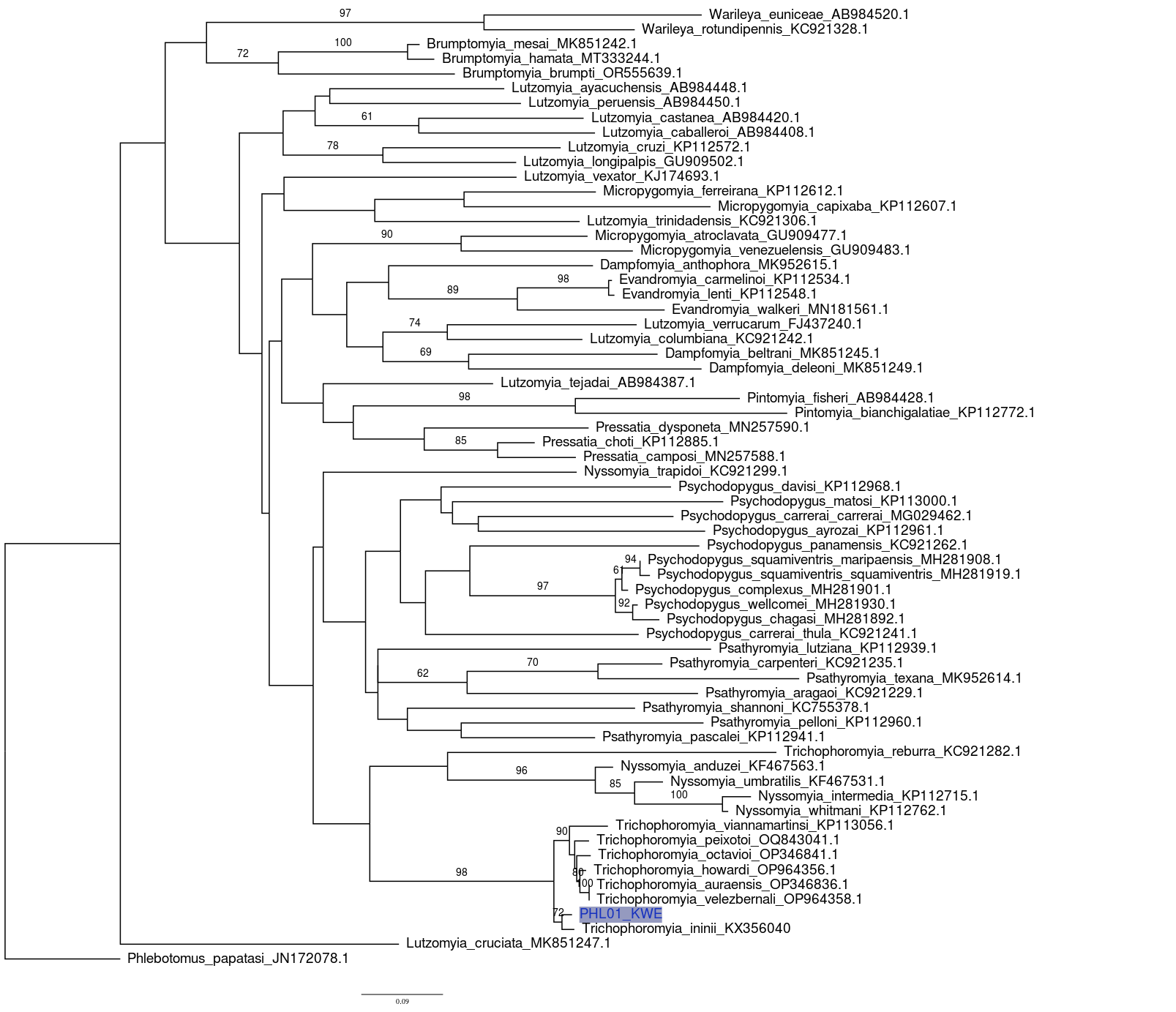


Figure S6. Maximum likelihood phylogeny of the sand fly *T. ininii* collected and sequenced at the Kwebana field site (labeled in blue). Tree was inferred using *COI* gene consensus sequences and based on 1,000 bootstrap replicates. Nodes with bootstrap support values of greater than 60 are listed. Label identifiers for comparative sequences (black labels) represent NCBI Genbank accession numbers.
